# Yeast-based evolutionary modeling of androgen receptor mutations and natural selection

**DOI:** 10.1371/journal.pgen.1010518

**Published:** 2022-12-02

**Authors:** Haoran Zhang, Lu Zhang, Shaoyong Chen, Mingdong Yao, Zhenyi Ma, Yingjin Yuan

**Affiliations:** 1 Frontier Science Center for Synthetic Biology and Key Laboratory of Systems Bioengineering (Ministry of Education), Tianjin University, Tianjin, People’s Republic of China; 2 School of Chemical Engineering and Technology, Tianjin University, Tianjin, People’s Republic of China; 3 Hematology-Oncology Division, Department of Medicine, Beth Israel Deaconess Medical Center and Harvard Medical School, Boston, Massachusetts, United States of America; 4 School of Basic Medical Sciences, Tianjin Medical University, Tianjin, People’s Republic of China; East China Normal University, CHINA

## Abstract

Cancer progression is associated with the evolutionary accumulation of genetic mutations that are biologically significant. Mutations of the androgen receptor (AR) are associated with the development of prostate cancer (PCa) by responding to non-androgenic hormones, and the lack of annotations in their responsiveness to hormone ligands remains a daunting challenge. Here, we have used a yeast reporter system to quickly evaluate the responsiveness of all fifty clinical AR mutations to a variety of steroidal ligands including dihydrotestosterone (DHT), 17β-estradiol (E2), progesterone (PROG), and cyproterone acetate (CPA). Based on an AR-driven reporter that synthesizes histidine, a basic amino acid required for yeast survival and propagation, the yeast reporter system enabling clonal selection was further empowered by combining with a random DNA mutagenesis library to simulate the natural evolution of AR gene under the selective pressures of steroidal ligands. In a time-frame of 1–2 weeks, 19 AR mutants were identified, in which 11 AR mutants were validated for activation by tested steroidal compounds. The high efficiency of our artificial evolution strategy was further evidenced by a sequential selection that enabled the discovery of multipoint AR mutations and evolution directions under the pressure of steroidal ligands. In summary, our designer yeast is a portable reporter module that can be readily adapted to streamline high-throughput AR-compound screening, used as a PCa clinical reference, and combined with additional bioassay systems to further extend its potential.

## Author summary

Androgens are a group of steroid-type sex hormones that regulate secondary sexual characteristics through interacting with the androgen receptor. Androgen-activated androgen receptor has been linked to prostate cancer progression, thus steroid drugs structurally similar to but different from androgens are clinically used to block androgen activation and to treat prostate cancers. However, mutations of the androgen receptor occur in clinics. As a result of some of these mutants, the steroidal hormone drugs become inactive and even by themselves can activate the androgen receptor exacerbating the disease conditions. Due to lack of an efficient screening method, how other clinical mutants respond to various steroidal hormone drugs remain unknown. Here, we developed a yeast-based system that allows high throughput study of the interaction between each hormone drug and androgen receptor mutant by eye-ball observation of yeast colony formation. Using this method, we thoroughly investigated all 50 clinically relevant mutants of the androgen receptor and how they individually respond to each of a variety of hormone drugs. Furthermore, the capacity of this system allowed us to perform random mutagenesis and quickly accumulate high volume of data regarding a large number of mutants, informative in guiding future precision medicine to treat prostate cancer.

## Introduction

Cancer progression is an evolutionary process in association with stepwise mutations conferring the advantages of cell survival and proliferation [1–6] Intratumor heterogeneity (ITH) refers to the co-existence of cell populations with genetic variations and distinct phenotypic profiles within tumors [7–9], serving as a driving factor in cancer progression and an important clinical barrier in precision medicine [10,11]. The Cancer Genome Atlas (TCGA) and International Cancer Genome Consortium (ICGC) data portals comprise extensive information on tumor genetic abnormalities, including point mutations, indels, and chromosome rearrangements, providing opportunities for advances in precision medicine [12,13]. However, most mutations recorded in databases are nonrecurrent and need systematic strategies to streamline their functional annotation [14]. Moreover, new inclusions to cancer mutation databases are restrained by the limited accessibility to patient specimens and the low mutation rate during cancer progression [15]. Hence, we focus on developing a yeast-based high-efficiency screening pipeline in conjunction with synthetic biology to simulate the random mutagenesis and natural selection of cancer-associated genes.

AR is a nuclear hormone receptor that plays a central role in prostate cancer tumorigenesis and therapy [16–18]. Aberrant AR activation has also been implicated in the pathogenesis of breast carcinoma [19] and hepatocellular carcinoma [20]. As a classic inducible transactivator, AR binding to androgenic hormones (such as testosterone and DHT) triggers its nuclear translocation and specific chromatin occupancy, driving a transcriptome network functioning in cell central metabolism and cell cycle progression [21,22]. The selection pressure of drugs on the AR pathway in PCa is evidenced by the enrichment of AR mutations in the ligand-binding domain (LBD) that broadens its ligand specificity spectrum to low levels of circulating androgens or even non-androgenic therapeutic ligands, reducing clinical treatment effects of PCa and the quality of patient survival [23–27]. Therefore, deciphering phenotypic consequences of clinical AR mutants in response to a variety of endogenous and therapeutic ligands helps to provide useful insights into the improvement of PCa treatments by understanding the genetic predispositions to PCa development and severity between individuals in therapies. However, a majority of recorded AR-LBD mutants in AR gene mutations database (ARDB) are of unknown functions, leaving gaps for applications in personalized medicine and clinical reference [28].

A yeast reporter system (*Saccharomyces cerevisiae*) previously developed by us [29] was employed here to carefully evaluate the transcriptional reactions of 50 clinical AR mutants recorded in ARDB, ranging from loss to gain of function in response to steroidal ligands. Given the limited number of PCa-relevant mutants documented in ARDB, combining our synthetic biology platform with this designer yeast, an artificial screening method was developed to imitate the natural evolution of AR under the selective pressure of various steroidal ligands. Novel AR mutants generated across the artificial evolution by random mutagenesis were carefully evaluated in our yeast system. In the initial round of screening, we identified 11 gain-of-function AR mutants with activated responsiveness to non-androgenic ligands, in which 6 were novel mutations and the other five belonged to the 56 known AR-LBD mutations chronicled in a 22-year period (from 1990 to 2012) [28]. A sequential AR evolution assay was conducted using the initial round mutants as founder templates, leading to the identification of multipoint AR mutations with enhanced responsiveness to ligands. By simulating the evolution and natural selection of AR using this yeast system, we are able to quickly get the information of a diversity of AR mutants in their ligand-responsiveness to different steroidal drugs, which may help to uncover novel insights into therapeutic development and precise medicine in the future. Collectively, our platform can be viewed from an evolutionary engineering prospect, and by linking the yeast reporter module to additional bioresources, it can be used to streamline the functional annotation of clinical AR mutants, screen AR compounds, simulate AR molecule natural evolution, and serve as a PCa clinical reference.

## Description of the method

### Yeast strains and mammalian cell lines

*S*. *cerevisiae* YRG-2 (Mata *ura3-52 his3-200 ade2-101 lys2-801 trp1-901 leu2-3 112 gal4-542 gal80-538* LYS2:UAS_GAL1_-TATA_GAL1_-HIS3 URA3:UAS_GAL4 17mers(x3)_-TATA_CYC1_-lacZ) was cultivated in YPAD medium (10 g/L yeast extract, 20 g/L peptone, 0.1 g/L adenine hemisulfate salt and 20 g/L dextrose). YRG-2 transformants co-harboring AR-LBD mutants and SRC-1 were cultured in synthetic complete medium (6.7 g/L yeast nitrogen base w/o amino acids, 2 g/L synthetic dropout mix, 0.1 g/L adenine hemisulfate salt and 20 g/L dextrose) without tryptophan and leucine supply (SC-Trp-Leu). The *Hep3B* cell lines were cultured at 37°C and 5% CO_2_ in Dulbecco’s Modified Eagle Medium (DMEM) supplied with 10% charcoal-stripped fetal bovine serum (CSS).

### Yeast assays

This is a modification of a previously described yeast assay [29]. In brief, YRG-2 transformants were grown to log phase in liquid medium SC-Trp-Leu at 30°C by overnight shaking. The overnight yeast cultures were harvested and washed twice with sterile water. The optical density of overnight yeast cultures at 600 nm (OD_600_) was measured in 96-well plates by a SpectraMax M2e plate reader (Molecular Devices). For liquid yeast assay, yeast cultures were diluted with synthetic complete medium without tryptophan, leucine and histidine supply (SC-Trp-Leu-His) to a cell density roughly at 4 ×10^4^ cells per milliliter (equivalent to an OD_600_ value of 0.002). Steroidal ligands purchased from Sigma-Aldrich (DHT, E2, PROG, CPA) were dissolved in ethanol and supplemented into 200 μL of diluted yeast cultures at indicated concentrations. Ligand-stimulated yeast cultures were then subjected to a static cultivation at 30°C in 96-well plates (200 μL of yeast cultures per well). OD_600_ values were measured 21–60 hours post-incubation. For plate yeast assay, diluted liquid yeast cultures with an initial cell density of 10^6^ cells per milliliter (equivalent to an OD_600_ value of 0.05) were subjected to ten-fold serial dilutions. Five microliters of each dilution were spotted on SC-Leu-Trp-His agar plates supplemented with appropriate steroidal ligands. Plates were photographed 2–3 days after static incubation at 30°C. For most liquid and plate yeast assays, an addition of 25 mM of 3-amino-1,2,4-triazole (3-AT) was added appropriately to the medium to inhibit the leaky expression of HIS3.

### Site-directed mutagenesis

Site-directed mutagenesis on AR-LBD, and the construction of plasmids expressing cytomegalovirus (CMV) promoter-regulated full-length AR mutants for mammalian cells were performed as previously described [29].

### Simulation of natural AR evolution against steroidal ligands

The library of mutagenized AR-LBD fragments was generated *via* error-prone polymerase chain reaction (PCR) with primers LBD-Library-F (5’-taa caa agg tca aag aca gtt gac tgt atc gcc ggg atc cgc ccg gaa gct gaa gaa act-3’) and LBD-Library-R (5’- taa atc ata aga aat tcg ccc gga att agc ttg gct gca gtc act ggg tgt gga aat aga-3’). Plasmids containing wild-type AR-LBD (pBD-AR_LBD) and Taq DNA polymerase purchased from Tiangen were employed in the error-prone PCR as DNA templates and DNA polymerases, respectively. Approximately 0.8 mM of Mn^2+^ was added to the Taq DNA polymerase buffer to achieve the highest single-point-mutation rate in each PCR product. The mutagenized PCR fragments were digested with *BamH*I and *Pst*I and inserted into the same sites in backbone plasmids, pBD-GAL4-cam. The ligation products were transformed into *E*. *coli*. Then, plasmid libraries harboring various AR mutants were isolated from pools of *E*. *coli* transformants, and co-transformed with SRC-1-expresing plasmids, pGAD424-SRC1, into YRG-2 stains. The library of yeast clones was selected on SC-Trp-Leu-His agar plates supplemented with appropriate steroidal ligands without the presence of 3-AT. Surviving clones were respectively subjected into an overnight cultivation in liquid SC-Trp-Leu medium with shaking. Due to the large number of clones, water-washed overnight cultures were directly diluted 100-fold with SC-Trp-Leu-His medium without the measurement and adjustment of OD_600_ values. Liquid yeast assays were then performed. Plasmids carried in yeast clones with high-ranking OD_600_ values were isolated and used as DNA templates in a PCR amplification of LBD region in AR genes. PCR products were then subjected to a Sanger sequencing to identify mutagenized AR-LBD fragments. Identified AR mutants were re-transformed individually into the parental designer yeast, YRG-2, together with plasmid pGAD424-SRC1. The responsiveness of these AR mutants to related steroidal ligands were finally determined using yeast assays in the presence of 3-AT. The simulation of second-round AR evolution was performed in the same process.

### Mammalian cell transfection and dual-luciferase assay

The assays were conducted as previously described with slight modifications [29]. The transfection of *Hep3b* cells was performed using Invitrogen Lipofectamine 3000 Transfection Reagent according to the manufacturer’s directions with a 3:1 ratio of regent to DNA (3 μL of Lipofectamine per 3000 μg of DNA). A day before transfection, *Hep3B* cells were seeded in 24-well plates at a density of 10^5^ cells/well in DMEM containing 10% CSS. Four hours before transfection, the medium was replaced with DMEM containing 2% CSS to eliminate any possible disturbance generated by serum hormones. A plasmid expressing firefly luciferase under the control of an androgen-responsive promoter mouse mammary tumor virus (MMTV-*LUC*), pGL3-MMTV-LUC, was co-transfected into *Hep3B* cells with plasmids expressing full-length (FL) AR and plasmids expressing *Renilla* luciferase (pRLTK), an internal control in the dual luciferase assay. The medium was replaced with fresh DMEM containing 2% CSS 6 hours after transfection. The vehicle regent (ethanal) and steroidal ligands were added to the medium 24 hours post-transfection. Cells were harvested 48 hours post-transfection, washed twice with phosphate-buffered saline (PBS) and resuspended in passive lysis buffer supplied by the kit, Dual-Luciferase Reporter Assay System (Promega). The AR-responsive activities of firefly luciferase normalized to *Renilla* luciferase activities were obtained according to manufacturer’s instructions.

### In silico structural alignment and analysis

The three-dimensional structure of AR-LBD from Homo sapiens (hAR_LBD) in complex with various ligands was downloaded from the RCSB protein database to characterize the AR_LBD structures. hAR_LBD-DHT (PDB ID: 2ama) were selected in this study. In addition, complex structural alignment and analysis were performed with Pymol software.

## Verification and comparison

### Correlation in AR-mediated transcription readouts between designer yeast and mammalian reporter systems

**Fig 1:**
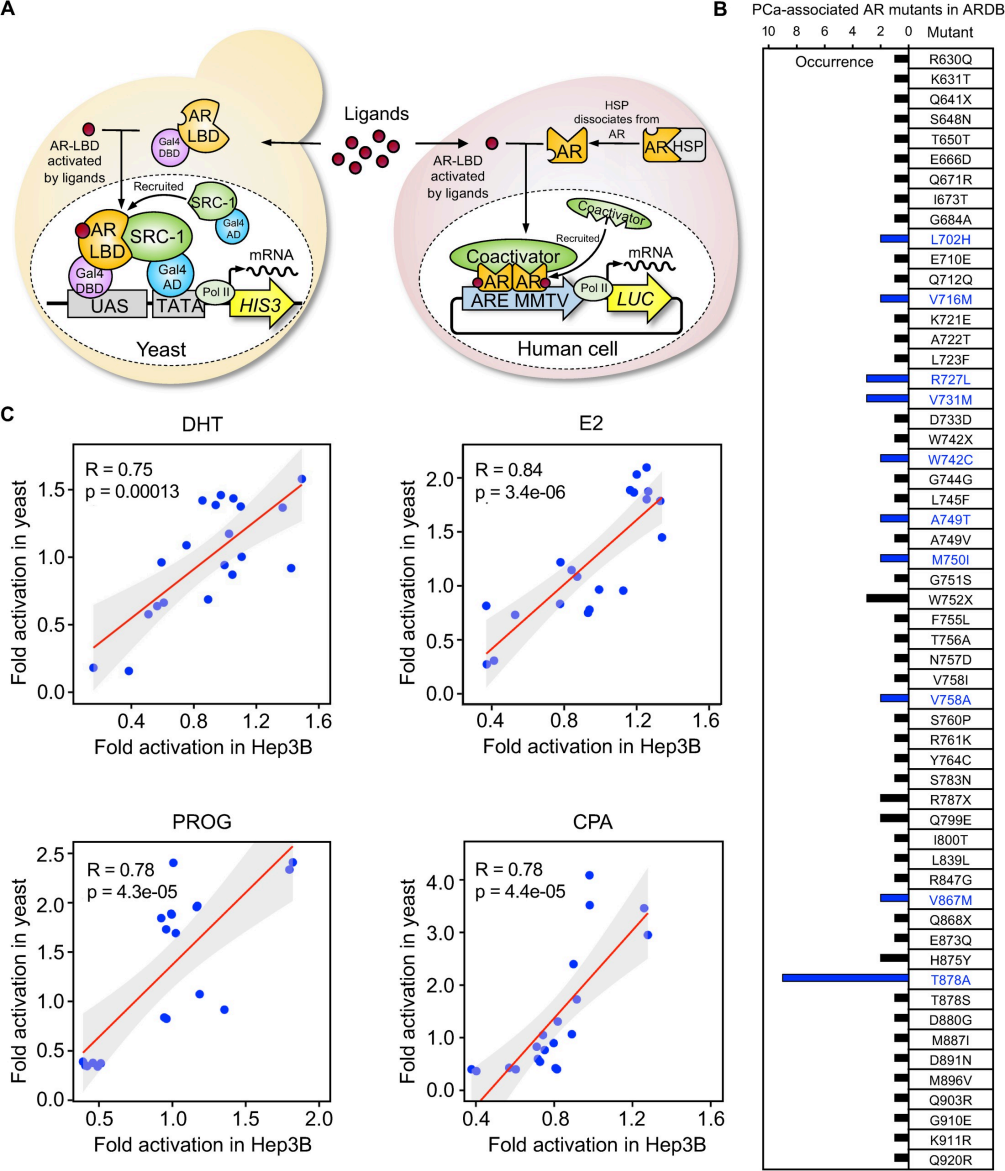
Correlation of AR-mediated reporter activities between designer yeast and mammalian systems. (**A**) Schematic representation of our designer yeast platform compared with the mammalian luciferase reporter system. (**B**) The list of 56 PCa-associated AR-LBD mutations accumulated in ARDB during a 22-year period (from 1990 to 2012). Top ten frequent mutations were marked in blue. (**C**), The Correlation analyses were based on fold changes in activation of AR-LBD mutants as normalized to the wild-type AR between the yeast and mammalian reporter systems, using the top ten frequent mutations in (**B**) against a variety of concentrations of DHT, E2, PROG, and CPA. Pearson’s correlation coefficient (R) and *P*-values were listed in the figures. Hormone concentrations in the yeast assay were DHT 10^−8^ M, E2 10^−5^ M, PROG 10^−5^ M, and CPA 10^−5^ M. Hormone concentrations in the human Hep3B reporter assay were DHT 10^−9^ M, E2 10^−7^ M, PROG 10^−9^ M, and CPA 10^−7^ M.

To gain insights into LBD mutation-directed and ligand-specific AR transactivation, we previously developed a GAL4-based yeast two-hybrid (Y2H) *HIS3* reporter system driven by the interaction between AR-LBD and steroid receptor coactivator-1 (SRC-1) that allows effective monitoring of the responsiveness of disease-associated AR mutants to clinical AR antagonists [29]. In the current study, we used this reporter to simulate AR gene evolution against a subset of steroidal compounds. The rationale of this designer yeast is based on the transactivation of the *HIS3* gene encoding histidine, an essential amino acid needed for yeast survival and propagation (Fig 1A). In comparison, an AR-dependent mammalian luciferase (*LUC*) reporter system [30–32] was developed by co-transfection of wild-type full-length AR (AR-WT) and MMTV-*LUC* reporter in human *Hep3B* cell lines lacking endogenous AR expression (Figs 1A and S1).

Next, we compared the reaction of clinical AR mutants to steroidal compounds in two reporter systems. During a 22-year effort from 1990 to 2012, 56 patient-derived AR-LBD mutations were identified and recorded in ARDB (Fig 1B) [28]; however, a majority (69%) of these mutants have not been functionally annotated. From this list, we selected 10 AR mutants with the highest clinical occurrence as highlighted in blue in Fig 1B (L702H [33,34], V716M [35,36], R727L [37,38], V731M [39,40], W742C [41,42], A749T [43,44], M750I [41,45], V758A [44], V867M [38,45], T878A [42,46]) to examine in both reporters. As shown in Fig 1C, an alignment of the LUC activity in *Hep3B* cells with the designer yeast growth profiling indicated a positive correlation in terms of responsiveness of mutants (as normalized to AR WT) to each specific compound (DHT [47], E2 [48], PROG [36], and CPA [49]). These findings support the consistency and reproducibility of these two systems. We also determined that the optimal compound concentrations are 10^−8^ M DHT, 10^−5^ M E2, 10^−5^ M PROG, and 10^−5^ M CPA in the yeast assay, and 10^−9^ M DHT, 10^−7^ M E2, 10^−9^ M PROG, and 10^−7^ M CPA in the mammalian system (S2 Fig). Differences in ideal compound concentrations could originate from multiple factors and suggested that both reporter systems possess unique features that could assist AR-directed compound development. Additionally, we have provided a summary of clinical cases related to these high frequency mutations in details describing the disease progression, severity, orchiectomy, and other medical treatments (Table 1). These patients were frequently diagnosed with advanced (metastatic) or androgen-independent prostate cancer and received orchiectomy and anti-androgen medications as treatments. In reality, the bulk of these high frequency mutations were found to be somatic mutations, which PCa patients most likely acquired while receiving the chemical therapy. In this circumstance, simulating the natural evolution of AR under the pressure of PCa-relevant chemicals becomes critical.

A plate assay demonstrated that even in the absence of any added steroidal ligand, a few out of 10,000 cells harboring the wild type AR-LBD (LBD-WT) were able to form visible colonies presumably due to leaky expression of HIS3 (S3 Fig). Such ligand-independent growth by no doubt would cause variables in the liquid assay. 3-amino-1,2,4-triazole (3-AT), a His3 enzyme (imidazoleglycerol-phophate dehydratase) inhibitor that has been used to avoid misinterpretation of data caused by leaky expression [50] was adapted into our system. Addition of 25 mM 3-AT abolished the colony formation due to leaky expression, while the activation effects of steroidal ligands on the LBD-WT and AR mutants sustained (S3 and S4 Figs).

**Fig 2:**
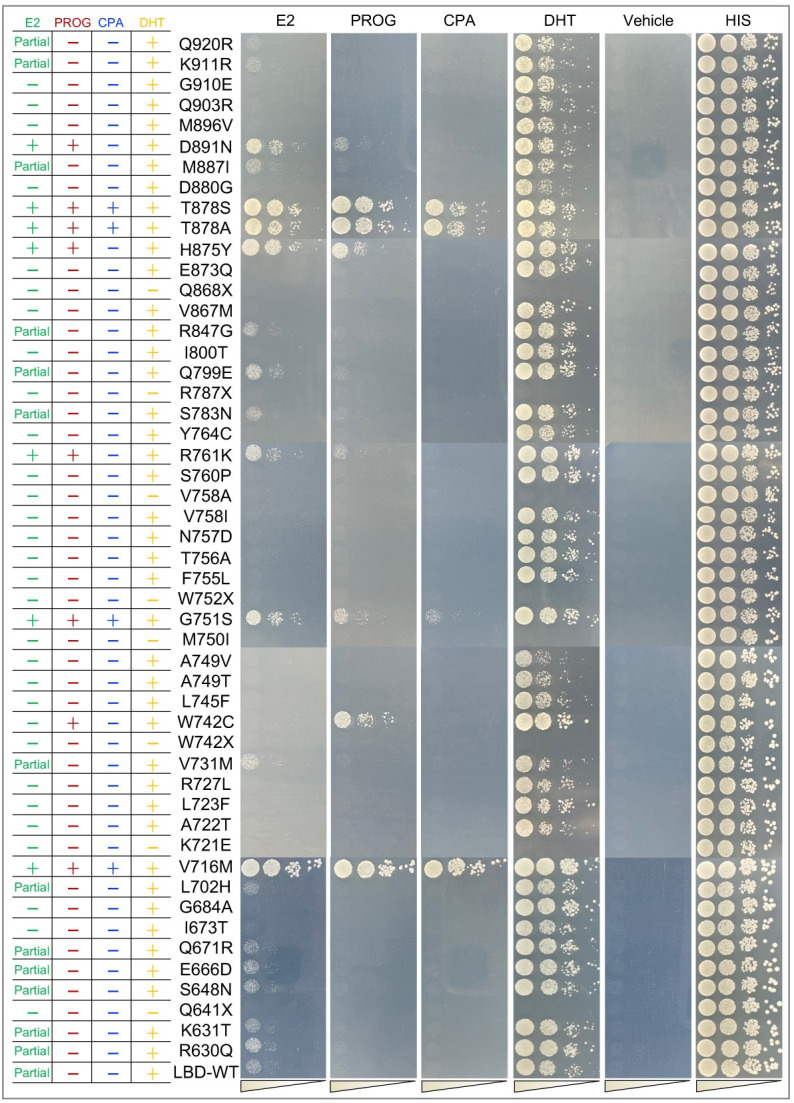
The functional analysis of 50 clinical AR mutants recorded in ARDB in response to steroidal ligands. The “+” mark indicates a full response of the AR mutants to indicated ligands was observed in plate yeast assays compared with LBD-WT, while the “–” mark indicates the opposite. The “Partial” mark indicated a partial ligand-induced response of the AR mutants. The concentration of all ligands in the yeast assay were 2 μM. Plates were photographed 60 h after incubation. Thirteen mutants (R630Q, K631T, S648N, E666D, Q671R, L702H, V731M, S783N, Q799E, R847G, M887I, K911R and Q920R) behaved similarly to the wild type protein showing full response to the physiological ligand DHT and partial response to a high dose (2 μM) of E2. Eight mutants (Q641X, K721E, W742X, M750I, W752X, V758A, R787X, Q868X) were identified as loss-of function mutations failed to respond to any tested steroids. Mutations ending in X like Q641X indicated that a stop-codon was generated in this position resulting in the expression of a truncated AR protein.

Among 56 clinically recorded AR mutations listed in Fig 1B, six are synonymous and are excluded from further investigation. The remaining 50 non-synonymous mutations were all subjected to careful evaluation using our yeast system, and the results are presented in Fig 2. Strikingly, mutants including V716M, G751S, T878A, and T878S responded to all four steroids. In addition to DHT, PROG was found capable of activating W742C, while both E2 and PROG, but not CPA, could activate three other mutants, R761K, H875Y and D891N. In addition, thirteen out of fifty mutants labeled as “Partial” exhibited only a partial response to high-dose E2 (2 μM) compared to the wild type AR (Fig 2). Eight mutants, Q641X, K721E, W742X, M750I, W752X, V758A, R787X, and Q868X, labeled as “-” under the DHT condition were identified as complete loss-of-function as they failed to respond to any tested steroids including the physiological ligand, DHT (Fig 2). Whether these mutants are expressed and folded properly remain to be investigated. Apart from these gain-of-function and loss-of-function AR mutants, 21 of the remaining responded to DHT only and are labeled as “+” under the DHT condition and as “-” under all the other conditions in Fig 2. Taken together, our system provided a convenient and quick assay to functionally characterize a variety of AR mutants in response to different steroidal ligands. As most of the mutants lack previous functional annotation and analysis, we could have compared it to the only available information of a few mutants in the literature and established the physiological relevance of our data. V716M was able to be trans-activated in response to PROG [36]. G751S, H874Y, T878S and D891N have been reported activatable by E2 and PROG [51,52] while T878A was reported responsive to E2 [48], PROG [48,53], and CPA [25]. K721E and V758A were reported to be inert to DHT [54]. All these previous findings were recaptured suggesting the reliability of our system.

## Applications

### Simulating the natural evolution of AR against steroidal ligands

**Fig 3:**
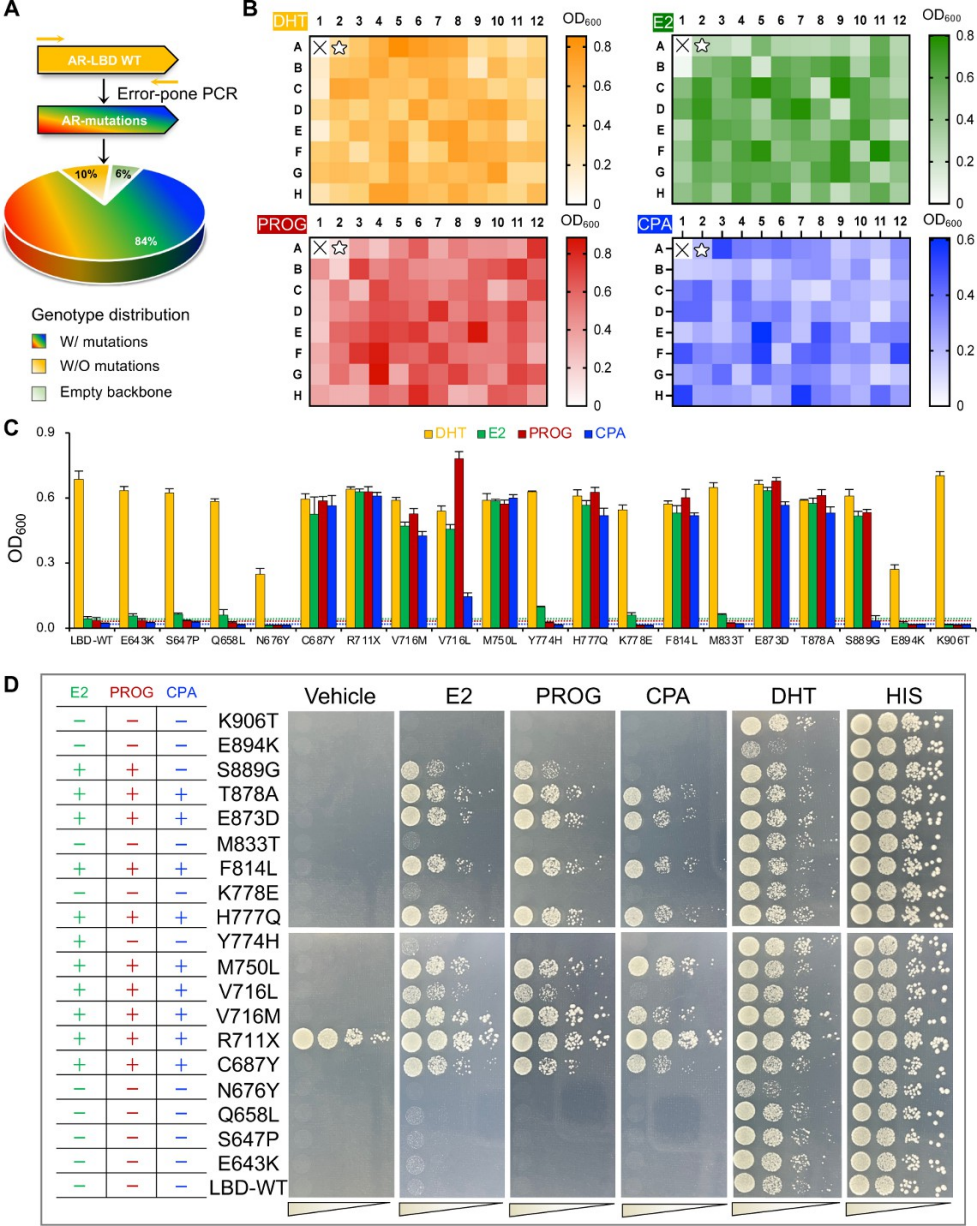
Simulation of AR evolution by combining designer yeast with random mutagenesis. (**A**) Characterization of the AR-LBD EP-PCR library. (**B**) Yeast clonal selection against compounds based on solid culture in 96-well plates without the presence of 3-AT. Ligands were 10^−8^ M DHT, 10^−5^ M PROG, 10^−5^ M E2, and 10^−5^ M CPA. The heatmap represents OD_600_ values of liquid cultures measured 21 h post-incubation. LBD-WT is indicated by pentacles; the × mark indicates blank wells. (**C**-**D**) Functional analysis of 19 identified AR mutations using both liquid (n = 6) and plate yeast assay in the presence of 3-AT against DHT 2 μM, E2 2 μM, PROG 2 μM, and CPA 2 μM. The incubation time was 60 h. Bars indicate mean ± s.d.

Next, we developed a strategy to artificially accelerate AR gene mutation and simulate its natural selection. For this purpose, we combined the designer yeast reporter system with error-prone polymerase chain reaction (EP-PCR) [55,56] to randomly synthesize an AR-LBD mutant library. The LBD mutant library was then transformed with SRC-1 into E. coli to obtain 10^6^ colonies. We isolated DNA from a pool of 100 random colonies and confirmed by targeted amplification and sequencing that the mutation rate of the LBD mutant library was 84% (Fig 3A). LBD-WT and empty backbone made up the remaining 16% of the library. Yeast carrying empty backbone plasmids did not grow in his-dropout media regardless of the presence of steroidal ligands, while yeast harboring the LBD-WT respond to DHT but not the other three steroidal compounds (S5 Fig). In order to obtain a broader range of candidate yeast colonies, the LBD mutant library was screened in designer yeast against each of the four ligands without the presence of 3-AT. And 94 colonies top-ranked in size were selected for further evaluation in 96-well liquid culture. As shown in the cell growth heatmaps, the selected yeast colonies exhibited variations in growth against distinct ligands, with a yeast colony bearing the WT AR-LBD as control (Fig 3B). Eventually, 12 top-scored yeast colonies based on liquid growth profiling were selected from each compound group, followed by DNA isolation and targeted amplification and sequencing.

**Fig 4:**
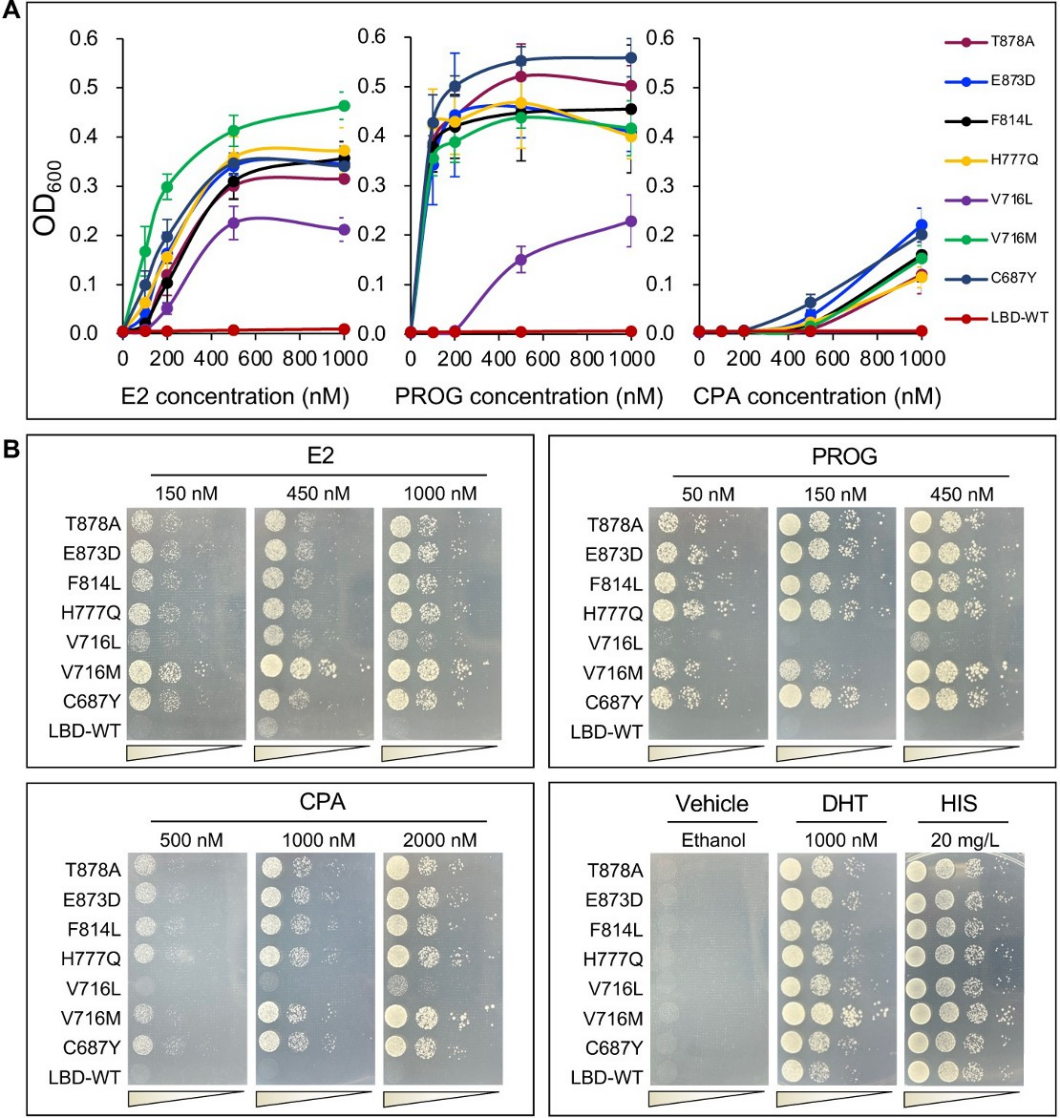
The evaluation of identified AR mutants in a dose-dependent yeast assay against steroidal ligands. (**A**) Liquid dose-dependent yeast assay with 25 mM 3-AT added (n = 5). OD_600_ values were measured 48 h post-incubation. Bars indicate mean ± s.d. (**B**) Plate dose-dependent yeast assay with 25 mM 3-AT added. Plates were photographed 48 h post-incubation.

As a result of the above AR simulation assay, 19 LBD single-point mutations that confer substantial yeast growth were identified. To clarify complications arising within the host under the pressure of long-term culture, we individually re-transformed each of these AR mutants into the parental designer yeast. With the presence of 25 mM 3-AT, we performed the liquid assay in 6 replicates as well as the plate assay on these AR mutants (Fig 3C and 3D). In comparison with LBD-WT, 11 out of the 19 mutants were verified to have superior AR transactivation activities in response to at least one steroidal ligand and marked with the + symbol in Fig 3D. And 5 out of these 11 mutants were reported clinically relevant to PCa by others (C687Y [57], V716M [58], M750L [59], T878A [60], and S889G [61]). In particular, V716M and M750L were reported to be trans-activated in response to PROG and E2, respectively [36,59]. And T878A was reported as an E2/PROG/CPA-responsive mutant [25,48,53]. The identification of these clinically relevant AR mutants from the mutation library further affirmed the reliability of our artificial evolution strategy. The remaining 6 of the 11 superior mutants were identified as novel AR mutants. Among them, the truncated R711X mutant constitutively exhibited transactivation activities which are independent of the ligand. The Y774H mutant conferred a weak response to E2 stimulation and was not activated by either PROG or CPA. Thus, we decided to focus on the remaining four novel mutants, V716L, H777Q, F814L, and E873D, and subjected them to the ligand-dose-dependent assays (Fig 4A and 4B). And three other clinical mutants (C687Y, V716M, T878A) selected from the mutant library were included as positive controls. The ligand-dependent mutant effects were further established by dose-dependent assays on these mutants (Fig 4A and 4B).

**Table 1:** Summary of clinical cases related to indicated AR mutations.

Mutations	Case descriptions of PCa-patients in previous studies	References
L702H	A patient with endocrine therapy-resistant prostate cancer, treated with castration and administration of chlormadinone acetate at stage D2.	[33]
L702H	A PCa-patient (stage D) refractory to hormone therapy.	[34]
V716M	A patient with androgen-independent prostate cancer.	[35]
V716M	A patient with advanced metastatic prostatic tumors, failing to respond to endocrine and cytotoxic therapy.	[36]
R727L	An untreated PCa patient with a germline AR mutation of R727L.	[37]
R727L	A PCa-patient treated with orchiectomy with a germline AR mutation of R727L.	[38]
V731M	A PCa-patient at stage B with no evidence of metastasis.	[39]
V731M	A patient with localized prostate cancer, treated by radical retropubic prostatectomy.	[40]
W742C	A patient with recurrent PCa, treated with orchiectomy and bicalutamide.	[41]
W742C	A patient with androgen independent prostate cancer, who relapsed after a combined therapy of androgen ablation and bicalutamide treatment.	[42]
A749T	A patient with metastatic prostate cancer.	[43]
A749T	A PCa-patient with a somatic AR mutation of A749T.	[44]
M750I	A patient with recurrent PCa, treated with orchiectomy and bicalutamide.	[41]
M750I	A PCa-patient with a somatic AR mutation of M750I.	[45]
V758A	A patient with metastatic prostate cancer carrying a somatic AR mutation of V758A.	[44]
V867M	A PCa-patient with a somatic AR mutation of V867M.	[45]
V867M	A PCa-patient with a somatic AR mutation of V867M, treated with orchiectomy.	[38]
T878A	A patient with androgen-independent prostate cancer, treated with flutamide, carrying a somatic AR mutation of T878A.	[42]
T878A	Two patients with advanced metastatic prostate cancer (bone metastasis): Both patients were at stage D2, treated with castration plus daily administration of 100 mg of chlormadinone acetate.	[46]

### Identification of three types of AR mutants in the artificial evolution assay

AR-LBD contains 12 helices: helix 3, 5, and 11 (red) envelop the ligand-binding pocket (LBP), and loops 1–3 (green) and 11–12 (green)/helix 12 (red) gate access to LBP [62]. Based on the crystal structure of DHT-bound AR-LBD (2ama) [63] and the positions of each specific mutation, we clustered these identified AR mutants into three types (Table 2). Type I mutation sites had direct engagement with ligands, Type II mutations were in proximity to LBP, and type III mutations were located normally more than 8 angstroms away from LBP. Considering the relative positions of these three types of mutants to LBP, we next compared their ligand-specific reactions in both reporter systems.

**Table 2:** Summary of known and novel AR-LBD mutations based on protein structure.

Type	Amino acid change	Location
**I**	M750L	H5
	M750I	H5
	T878A	H11
**II**	C687Y	Loop1–3
	L702H	H3
	V716M	H3
	V716L	H3
	R711X	H3
	V731M	H5
	W742C	H5
	A749T	H5
	V758A	H5
	V867M	H11
	E873D	H11
	S889G	Loop11–12
	E894K	Loop11–12
	K906T	H12
**III**	R727L	H4
	E643K	N-t-loop
	S647P	N-t-loop
	Q658L	N-t-loop
	N676Y	H1
	Y774H	H6
	H777Q	H6
	K778E	H6
	F814L	H8
	M833T	H9

**Fig 5:**
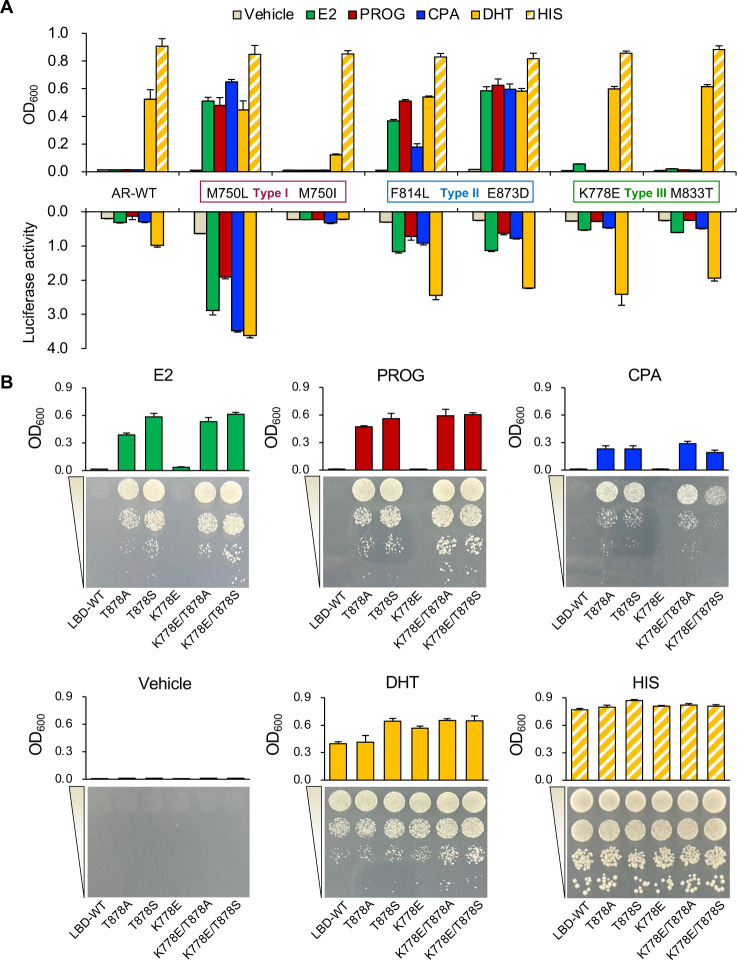
Sequential simulation of AR natural evolution. **(**A) Two representative mutations per type are shown in yeast assays (n = 6) and luciferase assays (n = 3). OD_600_ values were measured 60 h post-incubation. The concentration of each ligand was 2 μM for yeast assays and 10 nM for luciferase assays. (**B**) The assessment of double-mutations identified in the second-round evolution, K778E/T878A and K778E/T878S, against tested steroidal ligands in liquid (n = 6) and plate yeast assays (with 25 mM 3-AT added). The concentration of each ligand was 2 μM. OD_600_ values were measured 48 h post-incubation. Plates were photographed 48 h post-incubation. Bars indicate mean ± s.d.

For each LBD mutation type, two representative AR mutants were selected for parallel comparison in the two reporter systems to delineate their activation profiling against four ligands (Fig 5A). Interestingly, two type I mutants (AR-M750L and AR-M750I) exhibited opposite ligand-induced activities: AR-M750L was highly activated by DHT, E2, PROG, and CPA; whereas AR-M750I was less reactive to these four compounds. Both type II mutants (V716L and E873D) were gained in response to E2, PROG, and CPA. In contrast to LBD-WT, type III mutations K778E and M833T could be mildly triggered by E2. The minor response of the K778E and M833T mutants to E2 were easily caught by comparing the OD_600_ values of liquid yeast cultures (Figs 3C and 5A), but it was too minute to be observed with the naked eye in a plate yeast assay (Fig 3D). Despite the fact that we were able to identify 19 AR mutants in a single round of screening, more high-throughput screening tests will still need to be carried out in the future in order to examine the potential activation patterns of distinct types of AR mutant in response to diverse steroidal ligands. Whether these mutants could occur clinically and what biological functions they may have in regulation of cell proliferation and tumor development remain to be investigated in the further as well. Nevertheless, luciferase assays in *Hep3B* cells have been performed and the responses of all four new mutants K778E, F814L, M833T, E873D identified in this study to different steroids are fully consistent with the trend observed in the yeast assays (Fig 5A), valuing screening and preliminary functional analyses in the yeast system.

### A sequential artificial evolution to simulate the iterative natural selections of AR

Our yeast platform is designed to simulate the process of PCa progression driven by somatic AR mutations, and it simulates iterative AR natural evolution in a short period. Thus, we selected one founder mutant from each LBD mutation type (M750L from type I, E873D from type II, and K778E from type III) as the template for a second round of screening, which was again based on EP-PCR in conjunction with designer yeast. This iterative experimental design allowed assessing additional and multipoint LBD mutations that may shed light on the direction of AR gene evolution.

The mutation rates of the secondary AR-LBD mutant library based on EP-PCR were 84% for M750L, 86% for K778E, and 83% for E873D, respectively (S6 Fig). Once again, for each library, 94 best-ranked yeast colonies were selected against each compound, and the ligand responsiveness profiling was mapped (S6 Fig). For each secondary mutant library screening, a total of 48 colonies with 12 under each steroid condition (DHT, E2, PROG, CPA) with the highest activation scores were subjected to DNA isolation and sequencing. Table 3 summarizes the identified AR mutants. M750L, T878A and T878S that have been reported as clinical mutants were again among the results, suggesting the validity of the screening. Based on the readouts, there were no additional mutations on the M750L template in response to CPA, indicating that this mutant may have peaked in transactivation. However, both E873D and K778E secondary libraries acquired additional mutations that conferred even higher reaction capacity to ligands. We were not able to verify each of these AR mutants by re-transformation of it back into the parental designer yeast and only selected the mutants with the highest frequencies for subsequent analyses. In particular, the sequencing results of K778E and E873D secondary libraries (Table 3) revealed a considerable frequency of R711X, a truncated mutation that is constitutively active (Fig 3C and 3D), and other three E2/PROG/CPA-responsive mutations including M750L, T878A, and T878S (Figs 2 and 3C and 3D). Although the single-point mutation K778E exhibited only a low-level response to E2 and non-response to either PROG or CPA, the derived double mutations K778E/T878A and K778E/T878S have inherited the highly-responsive characteristics to all tested steroidal ligands from T878A and T878S mutations, respectively (Fig 5B). Data from additional luciferase assays for the secondary mutants K778E/T878A is consistent with the results of the yeast assays (S7 Fig). The above results further validated our yeast system in simulating the continuous evolution of AR.

**Table 3:** Summary of sequenced AR mutations acquired in the secondary mutation library.

Template	Condition	Mutants	Frequencies
M750L	E2	M750L/S648G	1
	E2	M750L/I738N	1
	E2	M750L/F795L	1
	E2	M750L/M887L	1
	E2	M750L/H918N	1
	E2	M750L/ F917S/H918N	1
	PROG	M750L/M735L	1
	PROG	M750L/Y858F	1
	PROG	M750L/D891H	3
	DHT	M750L/G910W	1
	DHT	M750L/L635P/F827Y	1
K778E	E2	K778E/R711X	1
	E2	K778E/R780W	1
	E2	K778E/T878S	1
	E2	K778E/K906M	1
	E2	K778E/Q712X/Q903L	1
	E2	K778E/Q712X/F755I/Q903L	2
	PROG	K778E/R711X	1
	PROG	K778E/S779T	1
	PROG	K778E/Q920P	1
	PROG	K778E/T878S	2
	PROG	K778E/Q903H	1
	PROG	K778E/R711X/K837E	1
	PROG	K778E/R711X/W797R	2
	PROG	K778E/Q712X/F755I/Q903L	1
	CPA	K778E/N706X	1
	CPA	K778E/E707G	1
	CPA	K778E/R711X/K837E	2
	CPA	K778E/R711X/K848X	1
	CPA	K778E/R711X/W797R	1
	CPA	K778E/M750L/D829E	1
	CPA	K778E/M750L/Q920P	1
	CPA	K778E/Q799R/T878A	1
E873D	E2	E873D/R711X	1
	E2	E873D/M750L	1
	E2	E873D/C845S	1
	E2	E873D/R856L	1
	E2	E873D/E707X/Q712L	1
	E2	E873D/M750L/K837X	2
	E2	E873D/R775L/A897V	1
	PROG	E873D/T878S	2
	PROG	E873D/R711X/T756S	2
	PROG	E873D/Q712R/T878A	1
	PROG	E873D/F857L/T878A	1
	PROG	E873D/Y740L/T878A/M887L	2
	PROG	E873D/Q734L/M743V/F877I/K884E/H886L	1
	CPA	E873D/R711X	1
	CPA	E873D/Y740N	1
	CPA	E873D/R711X/K911R	1
	DHT	E873D/W742R	1
	DHT	E873D/H918P	1

## Discussion

AR-LBD mutations altering ligand specificity are known drivers of PCa progression, leading to distant metastasis or therapeutic resistance [53,58,64–68]. However, the current mutation discovery and validation strategies are limited by the low mutation rate in clinical specimens and the lack of effective screening pipelines [16,69]. Consequently, inadequate functional annotation of cancer genome datasets restrains adequate bioresource usage, such as drug discovery and clinical references [26,70,71]. Indeed, PCa research faces challenges in overcoming hurdles in information flow and streamlining the functional interpretation of AR gene mutations. Here, we report that an integration of our designer yeast platform with synthetic biology would potentially fill the gap between AR-LBD digital discovery and precise PCa medicine.

This yeast reporter system is highly efficient, allowing clonal identification of single mutations generally not possible in mammalian reporter systems. The yeast platform can be used in both solid and liquid media formats, conferring convenience in data visualization and quantitation. Based on our designer yeast, we were able to score and rank responsiveness of AR mutants to distinct compounds. By combining our designer yeast assay with EP-PCR, we demonstrated its potential in high-throughput screening of mutants to steroidal compounds. In addition, this strategy enables simulation of iterative AR natural selection based on sequential artificial evolution screening. Together, our designer yeast platform can simulate AR natural selection and streamline the functional annotation of AR mutations in the public domain.

Notably, in a time-frame of roughly 1–2 weeks our system readily scored 19 AR-LBD missense mutations, in which 11 mutants individually validated by re-transformation highly reacted with ligands. Of significance, five verified LBD mutations have been recorded in clinical AR databases, accounting for about 10% cases that were chronicled over a 22-year period. One prominent clinical mutant identified in both initial and second round of screenings using our yeast system, AR-T878A, is a well-known PCa-relevant driver [72]. We also attempted to interpret the findings in protein structure, and interestingly, we observed enrichments of these LBD mutations in three distinct clusters by structural modeling. In addition, the designer yeast can be readily adapted for sequential simulation, and we found that one mutant (M750L) peaked in reaction to compounds, while two other mutants (E873D and K778E) can be further activated by acquiring additional mutations. Therefore, our designer yeast has the capacity to predict AR-LBD evolution directions against various compounds. Hence, the designer yeast offers a unique platform to systematically screen the AR-LBD mutant library against the compound library.

We esteem our designer yeast as a powerful and portable AR-LBD/compound screening module. The yeast system is unique by offering a clean background against complicated mammalian protein–protein interactions and by allowing clonal selection that is generally unachievable in mammalian reporter systems. Although this Y2H system can be biased by the interaction of LBD with a single AR co-activator (SRC-1), it confers high interaction stringency. Moreover, our yeast-based strategy can help bridge the gap between personalized medicine and ever expanding digital bioresources, as exemplified by genomic sequencing and protein structure computation datasets. The application potential of our designer yeast can be realized in combination with additional biological techniques and bioassay systems. With the system we developed in this study, we were able to perform multiple rounds of random AR mutations followed by selection for histidine auxotrophy in the presence of various steroidal compounds mimicking natural evolution of cancer cells under selection pressures by steroid medications. Thus, this system can be adapted to predict the continuous process of cancer development related to the accumulation of AR mutations in response to medical treatments and other clinically relevant challenges. Based on these rationales, we envision the integration of our method into PCa clinical practice and therapeutic references.

## Supporting information

S1 Fig Optimization of experimental conditions in AR-mediated *LUC* reporter assay. Mouse vas deferens protein (MVDP) promoter was used as a negative control for MMTV promoter. Bars indicate mean ± s.d.
(TIF)

S2 Fig Parallel alignments of designer yeast versus mammalian reporter assays. A preliminary dose-dependent test for Fig 1C based on the readouts of ten indicated AR mutants (WT as control) in response to steroidal ligands. The OD_600_ values of liquid yeast cultures (without the presence of 3-AT) were measured 21 h post-incubation. Bars indicate mean ± s.d. (n = 2).
(TIF)

S3 Fig A supplement of 25 mM 3-AT in growth medium inhibited the formation of yeast colony due to leaky expression of *HIS3*. Plate pictures were taken 48 h post-incubation.
(TIF)

S4 Fig A dose-dependent assay to determine the appropriate concentrations of tested ligands supplemented in the yeast medium in the presence of 25 mM 3-AT. Plate pictures were taken 48 h post-incubation.
(TIF)

S5 Fig The ligand-responsive differences of yeast clones that individually harbored backbone vector, LBD-WT, and T878A. T878A was a E2/PROG/CPA-responsive AR mutant (n = 4 in liquid yeast assays).
(TIF)

S6 Fig The distribution and abundance of secondary AR-LBD mutant library. Distribution of secondary AR-LBD mutant library based on M750L, K778E, and E873D templates, respectively. Again, 94 high-ranking yeast colonies were selected for each ligand, followed by DNA isolation and sequencing to identify additional mutations. Ligands: 10^−8^ M DHT, 10^−5^ M PROG, 10^−5^ M E2, and 10^−5^ M CPA. The heatmap represents the OD_600_ values of liquid yeast cultures measured 21 h post-incubation (without the presence of 3-AT).
(TIF)

S7 Fig A luciferase assay confirming the transactivation response of K778E/T878A double mutant to tested ligands. Bars indicate mean ± s.d. (n = 3).
(TIF)

S1 Data The plots of Fig 1C were created in R using the ‘ggpubr’ package. The R codes as well as the relevant numerical values were described in this .docx file.
(DOCX)

S2 Data All numerical values described in the main figures (Figs 3 and 4 and 5) and supplementary figures (S1 and S2 and S5 and S6 and S7 Figs) are listed in this .xlsx file. (XLSX)
