# Supplementary figures and images for: Yeast-based evolutionary modeling of androgen receptor mutations and natural selection
Source: PLoS Genet. 2022 Dec 2;18(12):e1010518. doi: 10.1371/journal.pgen.1010518 (PMC9718406; doi:10.1371/journal.pgen.1010518)

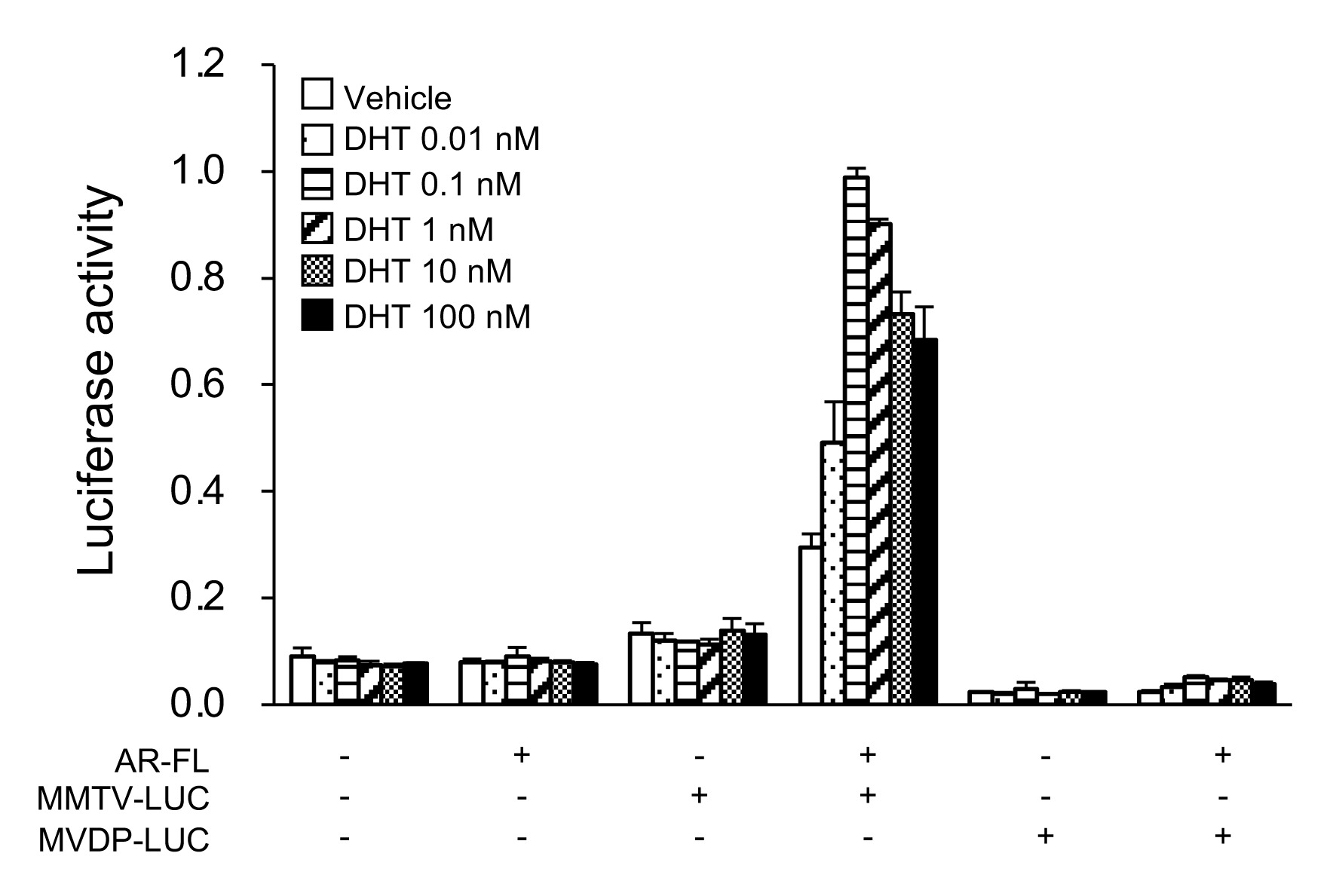

Supplement: S1 Fig — Mouse vas deferens protein (MVDP) promoter was used as a negative control for MMTV promoter. Bars indicate mean ± s.d. (TIF) [file pgen.1010518.s001.tif]

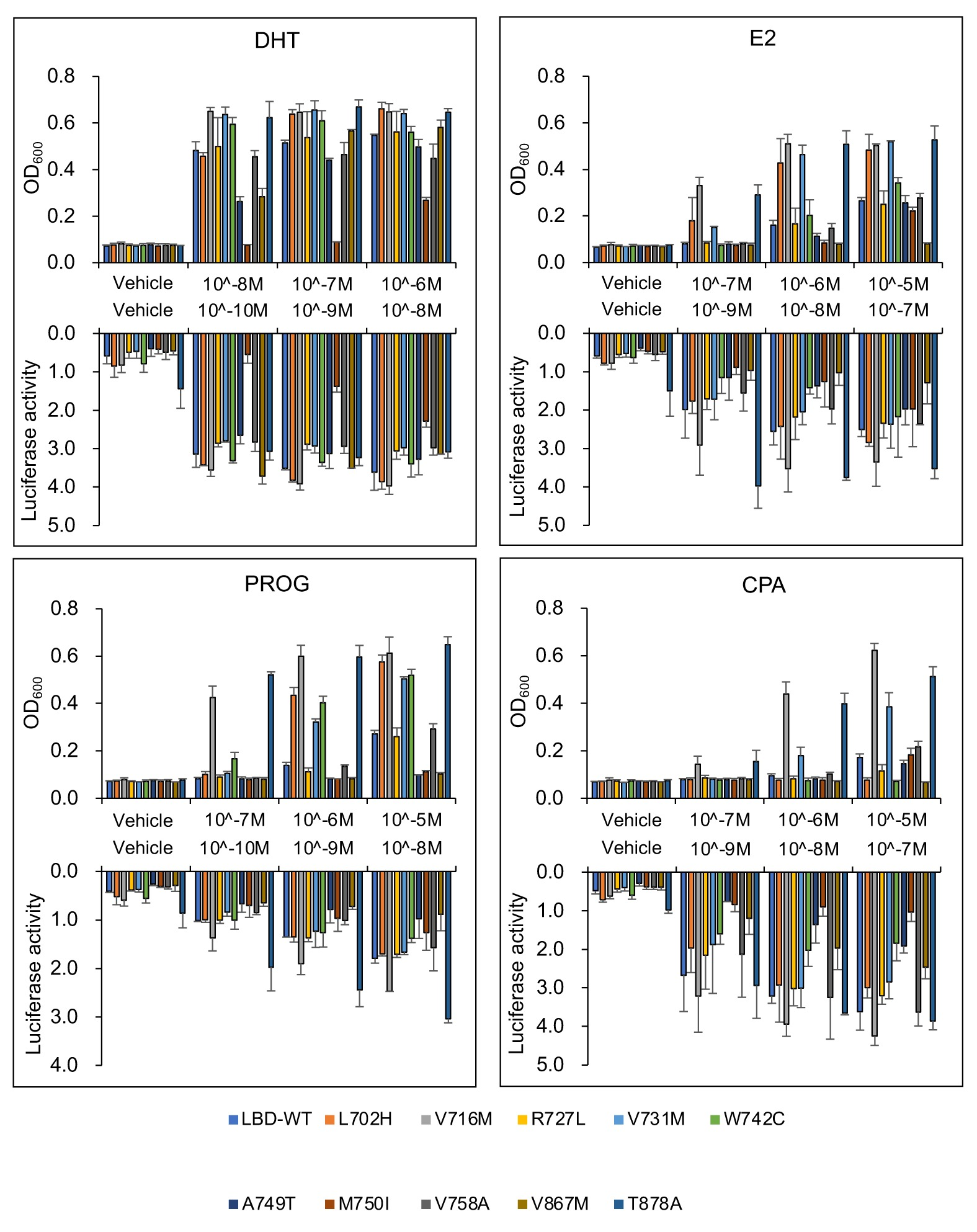

Supplement: S2 Fig — A preliminary dose-dependent test for Fig 1C based on the readouts of ten indicated AR mutants (WT as control) in response to steroidal ligands. The OD600 values of liquid yeast cultures (without the presence of 3-AT) were measured 21 h post-incubation. Bars indicate mean ± s.d. (n = 2). (TIF) [file pgen.1010518.s002.tif]

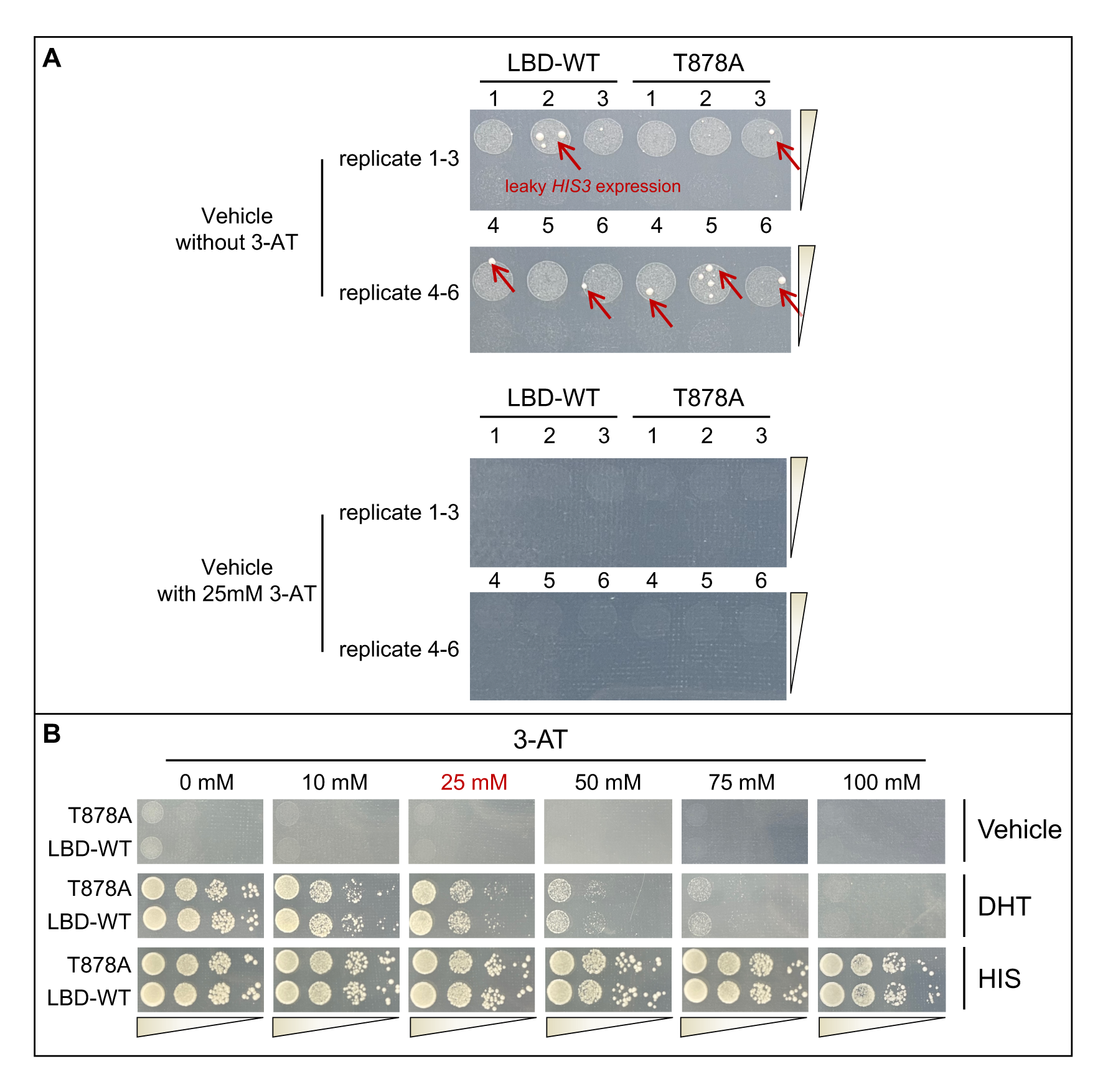

Supplement: S3 Fig — Plate pictures were taken 48 h post-incubation. (TIF) [file pgen.1010518.s003.tif]

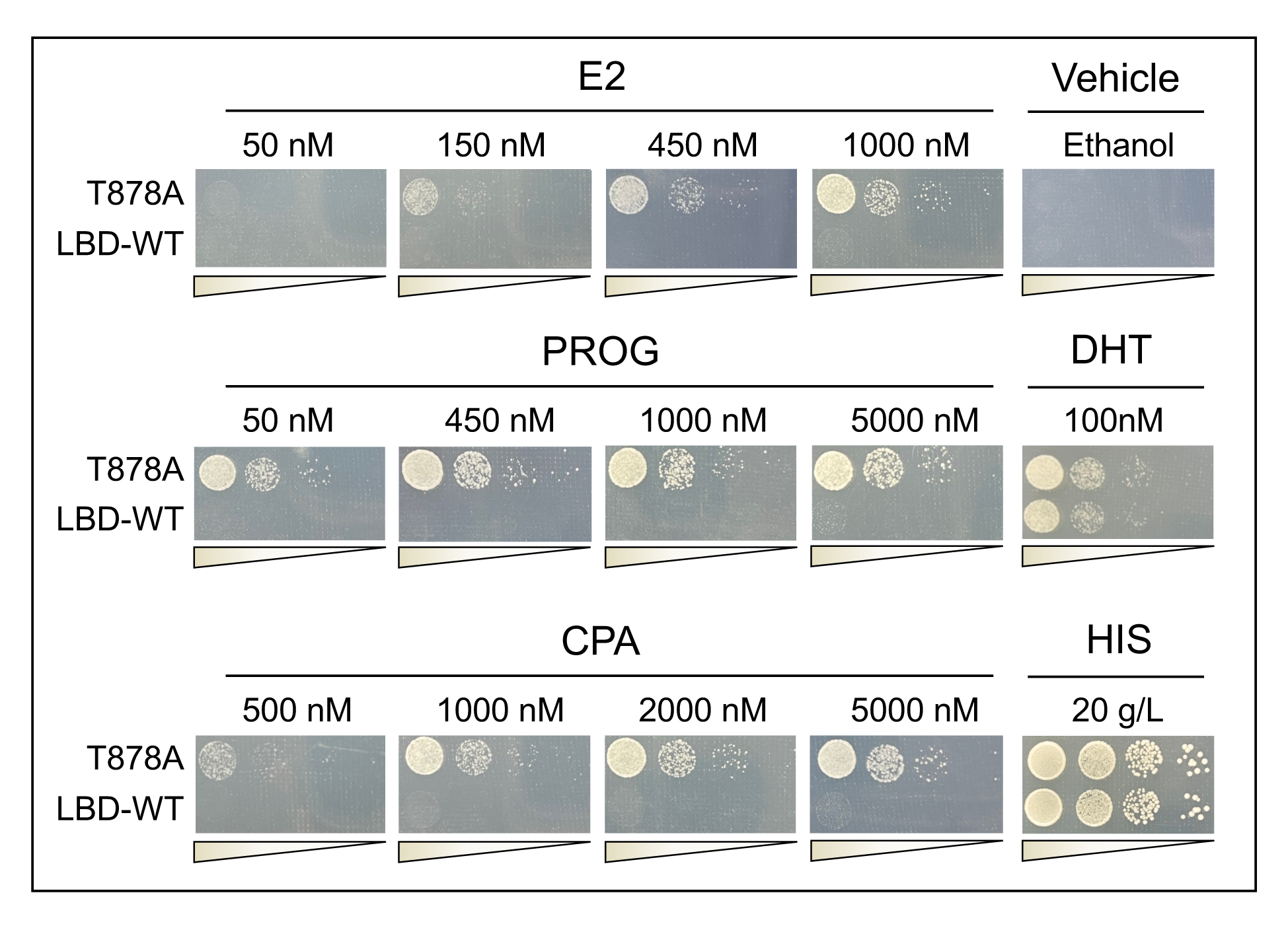

Supplement: S4 Fig — Plate pictures were taken 48 h post-incubation. (TIF) [file pgen.1010518.s004.tif]

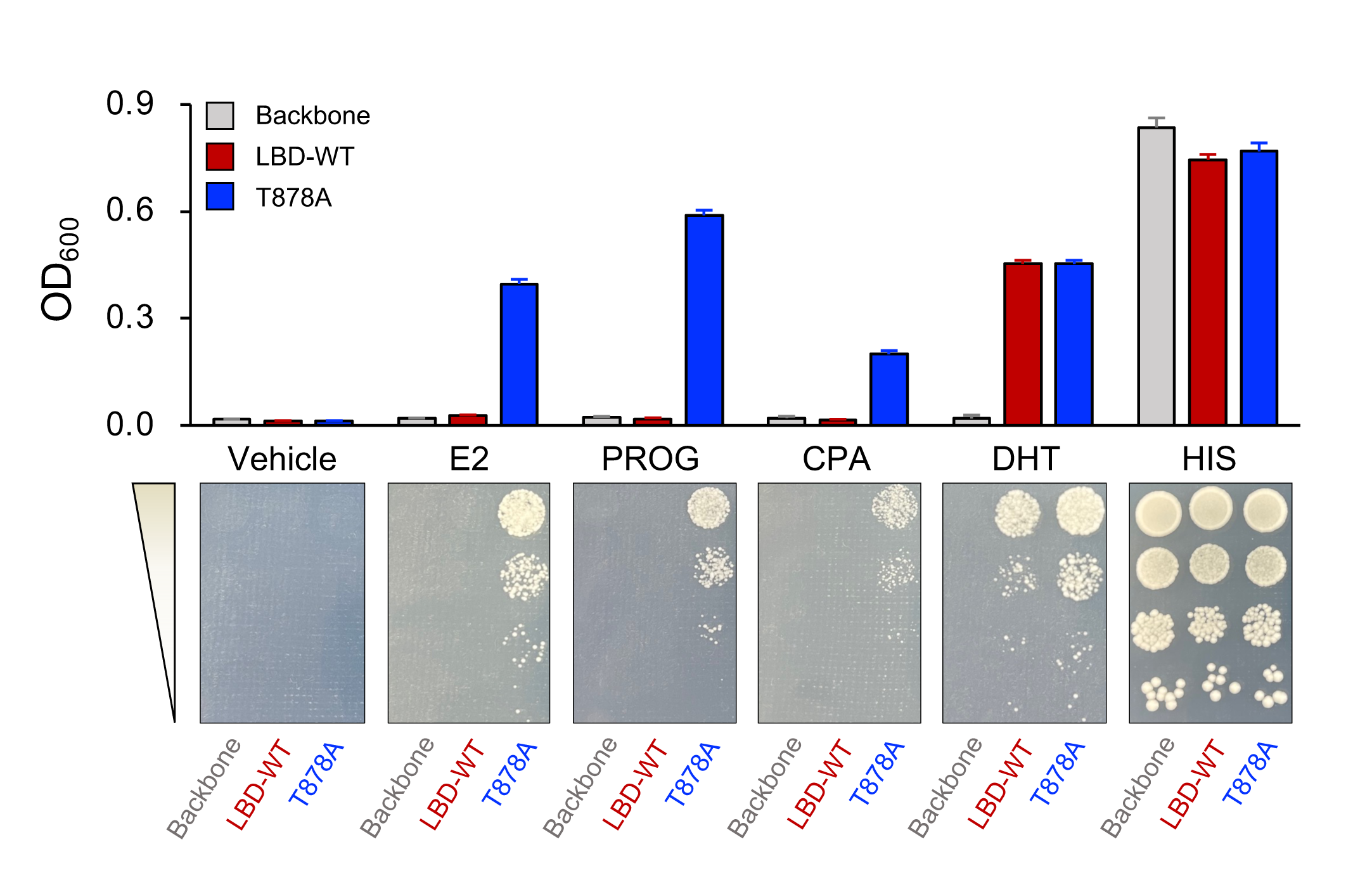

Supplement: S5 Fig — T878A was a E2/PROG/CPA-responsive AR mutant (n = 4 in liquid yeast assays). (TIF) [file pgen.1010518.s005.tif]

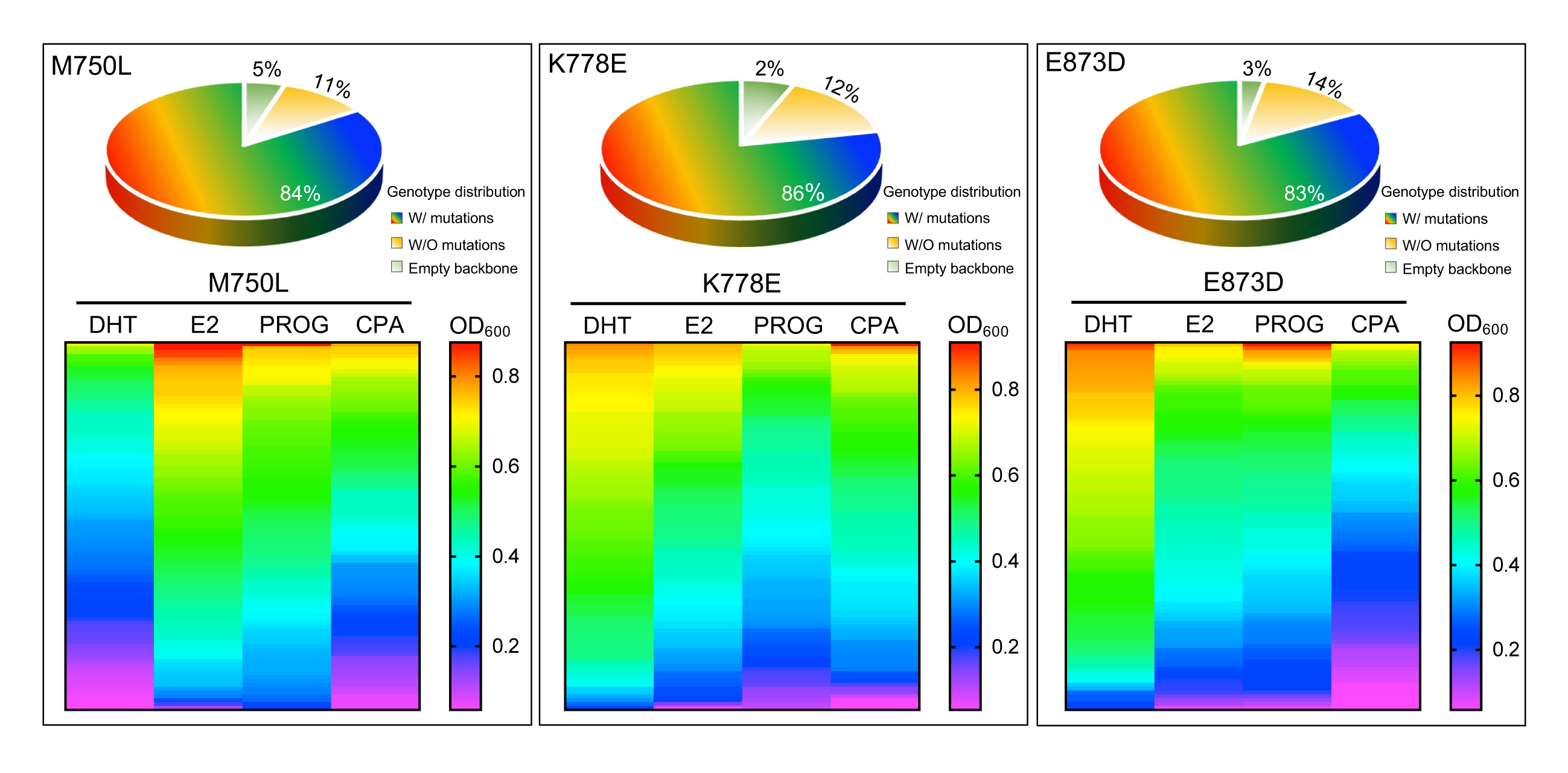

Supplement: S6 Fig — Distribution of secondary AR-LBD mutant library based on M750L, K778E, and E873D templates, respectively. Again, 94 high-ranking yeast colonies were selected for each ligand, followed by DNA isolation and sequencing to identify additional mutations. Ligands: 10−8 M DHT, 10−5 M PROG, 10−5 M E2, and 10−5 M CPA. The heatmap represents the OD600 values of liquid yeast cultures measured 21 h post-incubation (without the presence of 3-AT). (TIF) [file pgen.1010518.s006.tif]

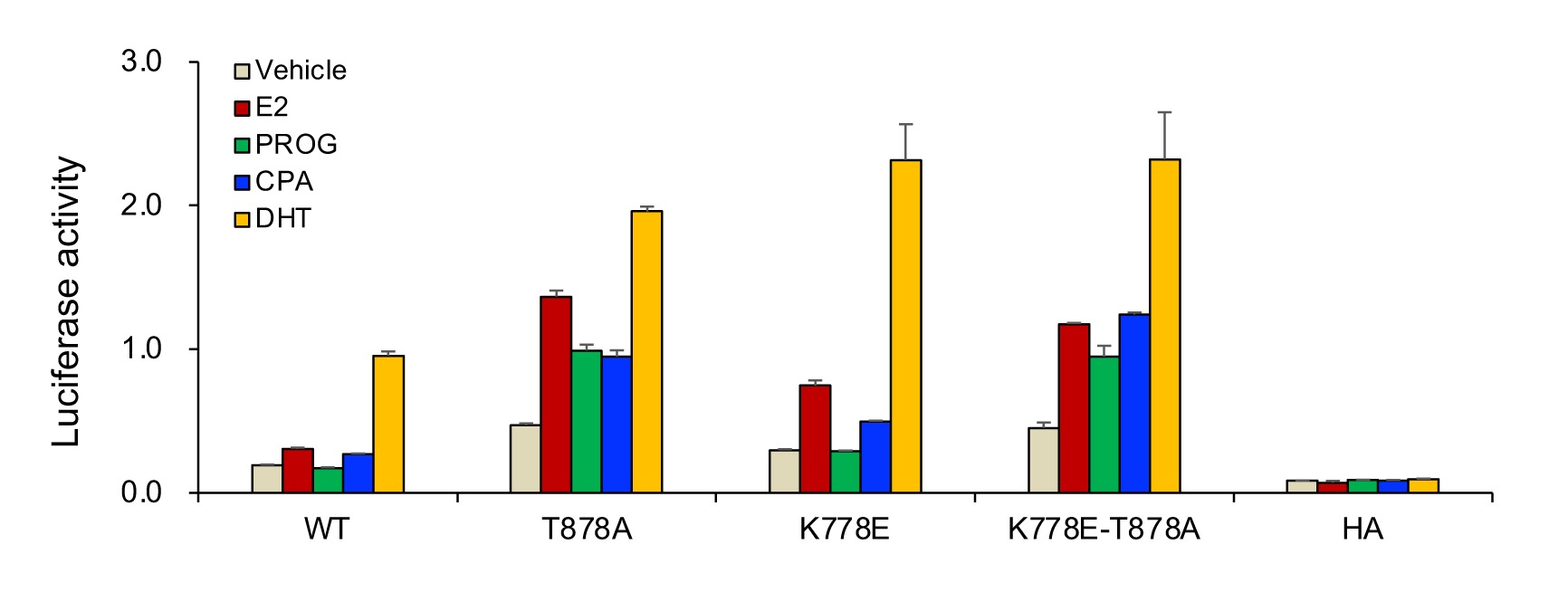

Supplement: S7 Fig — Bars indicate mean ± s.d. (n = 3). (TIF) [file pgen.1010518.s007.tif]
