# Supplementary material for: Yeast-based evolutionary modeling of androgen receptor mutations and natural selection
Source: PLoS Genet. 2022 Dec 2;18(12):e1010518. doi: 10.1371/journal.pgen.1010518 (PMC9718406; doi:10.1371/journal.pgen.1010518)
Supplement: S1 Data — The R codes as well as the relevant numerical values were described in this .docx file. (DOCX) [file pgen.1010518.s008.docx]

install.packages("ggpubr")

library("ggpubr")

library(scales)

xdht<-c(0.752916835,1.053092952,0.996325357,0.972913296,1.100331971,0.565443227,0.157447784,1.10595247,0.891379937,1.491515302,0.593012689,0.940028379,1.046809325,0.855220179,1.025287086,0.509374713,0.384115234,1.422946772,0.607431451,1.368532603)

ydht<-c(1.088190496,1.435316816,0.94161251,1.459534931,1.375203087,0.637794476,0.181204305,1.00307169,0.68719537,1.579356214,0.961438505,1.386410995,0.870501744,1.419985484,1.173374541,0.577298588,0.157102386,0.918756292,0.663365409,1.367165367)

dfDHT<-data.frame(xdht,ydht)

ggscatter(dfDHT,

x = "xdht",

y = "ydht",

color="blue",

size=6,

cor.method = "pearson",

add = "reg.line",

conf.int = TRUE,

fullrange = TRUE,

add.params=list(color="red",fill="lightgray",size=1.5),

cor.coef = TRUE,

cor.coeff.args = list(label.x.npc=0,label.y.npc=1,label.sep = "\n"),

cor.coef.size = rel(10),

xlab = "Fold activation in Hep3B",

ylab = "Fold activation in yeast",

title = "DHT",

xlim = c(0.1, 1.6),

ylim = c(0, 1.75),

ggtheme= theme_pubr(base_size = 32, border=TRUE) +

theme(axis.title=element_text(size=rel(1.1)),

plot.title=element_text(size=rel(1.1),hjust=0.5)))

xe2<-c(1.340001982,1.264263184,0.93183576,1.164348179,0.780071075,0.777674084,0.370220672,0.992954046,0.372741811,1.200947899,1.330405685,1.255779764,0.939428862,1.185170626,0.840834532,0.872154134,0.529419766,1.126068426,0.412917946,1.255220089)

ye2<-c(1.448600033,1.874450594,0.749959303,1.886741006,1.218500733,0.832370177,0.814585707,0.965204298,0.272790168,2.031458571,1.786418269,1.801762821,0.778605769,1.865024038,1.146233974,1.084735577,0.730849359,0.955769231,0.307051282,2.096995192)

dfe2<-data.frame(xe2,ye2)

ggscatter(dfe2,

x = "xe2",

y = "ye2",

color="blue",

size=6,

cor.method = "pearson",

add = "reg.line",

conf.int = TRUE,

fullrange = TRUE,

add.params=list(color="red",fill="lightgray",size=1.5),

cor.coef = TRUE,

cor.coeff.args = list(label.x.npc=0,label.y.npc=0.95,label.sep = "\n"),

cor.coef.size = rel(10),

xlab = "Fold activation in Hep3B",

ylab = "Fold activation in yeast",

title = "E2",

xlim = c(0.3, 1.6),

ylim = c(0, 2.2),

ggtheme= theme_pubr(base_size = 32, border=TRUE) +

theme(axis.title=element_text(size=rel(1.1)),

plot.title=element_text(size=rel(1.1),hjust=0.5)))

xprog<-c(1.165312412,0.995735167,0.945319594,0.924238557,0.991927954,0.407717504,0.391289308,1.186450041,0.420186881,1.797511316,1.170856886,1.006691899,0.960394657,0.958009703,1.023697308,0.490993201,0.513191888,1.354839135,0.458814566,1.820383757)

yprog<-c(1.956091159,1.879887046,0.838046375,1.843892932,1.888756314,0.350515054,0.38988983,1.073539355,0.345503719,2.335123096,1.968360349,2.404067955,0.824033666,1.730751247,1.691201683,0.3412952,0.373051746,0.916809539,0.378039277,2.409406172)

dfprog<-data.frame(xprog,yprog)

ggscatter(dfprog,

x = "xprog",

y = "yprog",

color="blue",

size=6,

cor.method = "pearson",

add = "reg.line",

conf.int = TRUE,

fullrange = TRUE,

add.params=list(color="red",fill="lightgray",size=1.5),

cor.coef = TRUE,

cor.coeff.args = list(label.x.npc=0,label.y.npc=0.95,label.sep = "\n"),

cor.coef.size = rel(10),

xlab = "Fold activation in Hep3B",

ylab = "Fold activation in yeast",

title = "PROG",

xlim = c(0.4, 2),

ylim = c(0.1, 2.5),

ggtheme= theme_pubr(base_size = 32, border=TRUE) +

theme(axis.title=element_text(size=rel(1.1)),

plot.title=element_text(size=rel(1.1),hjust=0.5)))

xcpa<-c(0.80701304,0.980447135,0.716309005,0.898470771,0.56989994,0.71063123,0.74250669,0.818165389,0.377410651,1.259226422,0.814147572,0.981607721,0.72740006,0.915350907,0.603466552,0.751239104,0.797266326,0.890180291,0.402265816,1.278534298)

ycpa<-c(0.421583569,4.087524494,0.596632557,2.397996952,0.427534654,0.828144278,1.050801945,1.307279193,0.399158139,3.461354235,0.399345822,3.517870949,0.542134999,1.727148379,0.398394291,0.7656854,0.896937258,1.06613143,0.364793339,2.952482902)

dfcpa<-data.frame(xcpa,ycpa)

ggscatter(dfcpa,

x = "xcpa",

y = "ycpa",

color="blue",

size=6,

cor.method = "pearson",

add = "reg.line",

conf.int = TRUE,

fullrange = TRUE,

add.params=list(color="red",fill="lightgray",size=1.5),

cor.coef = TRUE,

cor.coeff.args = list(label.x.npc=0,label.y.npc=1,label.sep = "\n"),

cor.coef.size = rel(10),

xlab = "Fold activation in Hep3B",

ylab = "Fold activation in yeast",

title = "CPA",

xlim = c(0.4, 1.6),

ylim = c(0, 4.5),

ggtheme= theme_pubr(base_size = 32, border=TRUE) +

theme(axis.title=element_text(size=rel(1.1)),

plot.title=element_text(size=rel(1.1),hjust=0.5)))
